# N-Acetyl-D-Glucosamine-Loaded Chitosan Filaments Biodegradable and Biocompatible for Use as Absorbable Surgical Suture Materials

**DOI:** 10.3390/ma12111807

**Published:** 2019-06-04

**Authors:** Milena Costa da Silva, Henrique Nunes da Silva, Rita de Cássia Alves Leal Cruz, Solomon Kweku Sagoe Amoah, Suédina Maria de Lima Silva, Marcus Vinícius Lia Fook

**Affiliations:** 1 Postgraduate Program in Materials Science and Engineering, Department of Materials Engineering, Federal University of Campina Grande, Campina Grande, PB 58429-900, Brazil; milecost@hotmail.com (M.C.d.S.); henrique.nunes@certbio.ufcg.edu.br (H.N.d.S.); 2 Department of Materials Engineering, Federal University of Campina Grande, Campina Grande, PB 58429-900, Brazil; ritaalvesleal@hotmail.com (R.d.C.A.L.C.); sulefan@gmail.com (S.K.S.A.); suedina.silva@ufcg.edu.br (S.M.d.L.S.)

**Keywords:** suture, chitosan, N-Acetil-D-Glucosamine, controlled release

## Abstract

The aim of this study was to prepare chitosan (CS) filaments incorporated with N-acetyl-D-Glucosamine (GlcNAc), using the wet spinning method, in order to combine the GlcNAc pharmacological properties with the CS biological properties for use as absorbable suture materials. The filaments were characterized by scanning electron microscopy (SEM), X-ray diffraction (XRD), uniaxial tensile testing, in vitro biodegradation, and through in vitro drug release and cytotoxicity studies. It was observed that the addition of GlcNAc did not alter the morphology of the filaments. The CS and CS/GlcNAc filaments presented diameters 145 µm and 148 µm, respectively, and the surfaces were homogeneous. Although the mechanical resistance of the chitosan filaments decreased with the incorporation of the GlcNAc drug, this property was greater than the mean values indicated in the U.S. Pharmacopeia (1.7 N) for suture number 6-0 (filament diameter of 100–149 μm). The biodegradation of the CS filaments was accelerated by the addition of GlcNAc. After 35 days, the CS/GlcNAc filaments degradability was at its total, and for the CS filaments it was acquired in 49 days. The in vitro kinetic of the release process was of the zero-order and Hopfenberg models, controlled by both diffusion and erosion process. The in vitro cytotoxicity data of the CS and CS/GlcNAc filaments toward L929 cells showed that these filaments are nontoxic to these cells. Thus, the GlcNAc-loaded CS filaments might be promising as absorbable suture materials. In addition, this medical device may be able to enhance healing processes, relieve pain, and minimize infection at the surgery site due the prolonged release of GlcNAc.

## 1. Introduction

Surgical sutures are filaments that represent one of the major categories of biomaterials for wound closure, both in human and veterinary medicine [1,2]. Filaments used in suturing can be classified according to the material used, the degradability, and the physical configuration (monofilament, multifilaments, twisted, or braided). They could be also impregnated or coated with drugs in order to improve their performance through drug direct administration [3,4,5,6]. 

The choice of the suture material used, with respect to its mechanical and biological properties, it is a factor dependent on the location and complexity involved in the healing wound process. The search for materials suitable for the wound specific necessity has led to sutures filaments development from natural and synthetic polymers [7,8]. Sutures of a natural origin are readily absorbed by the human body and dissolve more quickly than sutures of a synthetic origin. They may also have scar formation prevention, as well as properties such as being antimicrobial and antifungal [4,5,9]. Among those of a synthetic origin, are glycolic polyacid (Dexon), nylon (Ethilon™), polydioxanone (PDS II™), polyglactin 910 (Vicryl™), polypropylene (Prolene™) [10], and those from a natural origin are of catgut (collagen derived from intestinal submucosa from sheep), silk (from the silkworm *Bombyx mori * L.), cotton, and flax. In this sense, studies aiming to develop suture materials from natural biopolymers, such as collagen [11], chitin [12], and chitosan [13], have been conducted. Chitosan is a polymer derived from chitin, which has been found in a wide range of natural sources (the exoskeletons of the crustaceans, crabs, and shrimps, and the cell walls of fungi [14]) that exhibit biodegradability, biocompatibility, and hemostatic capacity, as well as the ability to inhibit the growth of microorganisms and accelerate the capacity of wound healing [15,16,17,18,19,20,21]. Besides non-toxicity and biocompatibility, chitosan can be degraded in vivo by several enzymes, mainly by lysozyme [22]. Furthermore, the products from degradation are non-toxic oligosaccharides, which can be then excreted or incorporated into glycosoaminoglycans and glycoproteins. All of these merits have made chitosan a suitable polymer to for medical and pharmaceutical applications [23,24,25,26,27,28]. 

Despite the benefits of chitosan, there are few studies reported in the literature about the use of this polymer for suture application, probably as a result of the difficulty of obtaining chitosan filaments with a suitable tensile strength [29]. Montenegro e Gordeiro [13] carried out a study in rats, using chitosan threads produced by the wet sppinning method, and confirmed the bacteriostatic activity of the suture threads. Viju e Thilagavathi [5] evaluated the antimicrobial activity in chitosan coating silk sutures, in which the suture that was treated with the highest concentration of chitosan presented an excellent antimicrobial activity. Gokarneshan e Dhatchayani [30] developed a silk suture coated with chitosan, and incorporated it with an extract from a natural Thermomyces fungus, which reduced the percentage of bacterial growth. 

At present, research and development efforts in sutures have mainly been aiming to absorbable materials, more specifically, those with multifunctionality, such as having antimicrobial, hemostatic, and biodegradability, and as devices for controlled drug release. As the polymer degrades, new diffusion pathways for the drug are created, improving the in situ delivery. On-site drug administration, by suturing directly in wound, provides an effient route for drug delivery [1,31,32,33]. Surgical sutures are biomaterials used in various procedures, which, when impregnated or coated with drugs, eliminate the need for other materials to be placed in the wound healing process [34]. However, to the best of our knowledge, up until now, no research about the manufacture of N-acetyl-D-glucosamine-loaded chitosan filaments for use as absorbable surgical suture materials has been carried out. 

N-acetyl-D-glucosamine (GlcNAc) is an amino monosaccharide obtained from chitin or chitosan by hydrolysis; in the literature, it is reported as the main component of the epithelium, playing essential roles in wound tissue repair. The administration of glucosamine in the first days after surgery can increase the production of hyaluronic acid in the wound, promoting a quick cicatrization, furthermore reducing the possible complications related to the process of tissue reconstruction [35,36,37]. Ashkani-Esfahani et al. [38] studied the effect of the topical administration of glucosamine on the wound healing process in rats, and found that the rate of wound closure, which is the main criterion for skin repair, was significantly increased, while the fibroblast proliferation and collagen synthesis also improved the revascularization process in the wound site. Because of its pharmacological properties and chitosan similarity, the incorporation of N-acetyl-D-Glucosamine into chitosan appears as a promising alternative to enhance the biological properties of chitosan filaments when applied in surgical sutures, which can accelerate the process of wound healing [39,40]. Thus, the aim of this study was to prepare chitosan (CS) filaments incorporated with N-acetyl-D-glucosamine (GlcNAc), using the wet spinning method, with the purpose of combining the pharmacological properties of GlcNAc with the biological properties of CS for use as an absorbable suture material. 

## 2. Materials and Methods

### 2.1. Materials

Medium molecular weight chitosan from the exoskeleton of *Lipopenaeus vannamei* shrimp (Mv = 270 kDa) determined by viscometry (PSL Rheotek, São Paulo, Brazil) [41], and the degree of deacetylation (DD = 88%) determined by the infrared spectroscopy (Perkin Elmer, Beaconsfield, U.K.) method [41] were produced in the Northeastern Biomaterials Evaluation and Development Laboratory—CERTBIO (Campina Grande, PB, Brazil). This chitosan (CS) is accredited by the National Institute of Metrology, Quality and Technollogy (INMETRO) at the International Organization of Standardization (ISO)/International Electrotechnical Commission (IEC) 17025:2005, and is used for medical applications. Lactic acid was obtained from Vetec^®^ (Duque de Caixas/Rio de Janeiro, Brazil). Sodium hydroxide (NaOH) and methanol (CH_3_OH) were purchased from Neon^®^ (São Paulo, Brazil). N-acetyl-D-glucosamine (>99%), phosphate buffered saline (PBS), and lysozyme (hen egg-white—HEW) were purchased from Sigma-Aldrich^®^ (Darmstadt, Germany). 

### 2.2. Fabrication of GlcNAc-Loaded CS Filaments

**Figure 1:**
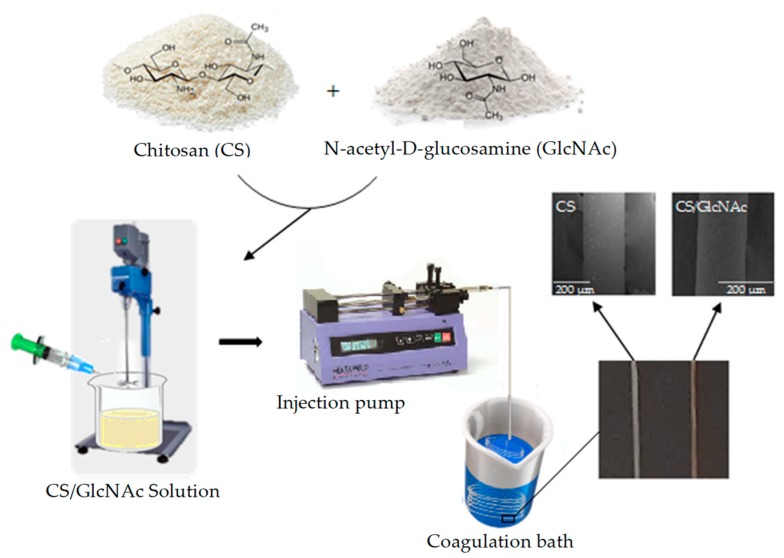
Schematic representation of the fabrication of chitosan (CS) and N-acetyl-D-Glucosamine (GlcNAc)-loaded CS wet-spun filaments.

The GlcNAc-loaded CS filaments were prepared using the wet-spun method. Firstly, the chitosan solution (4% *w*/*v*) [42,43] was prepared by dissolving the polymer in an aqueous solution of lactic acid, the latter being used stoichiometrically (0.21 mol/L) in relation to the amine groups of chitosan. The polymer solution was maintained under constant mechanical stirring at 600 rpm, at room temperature (25 ± 1 °C) for 2 h. After that, the GlcNAc (0.2 g) was added slowly to the chitosan solution (100 mL) by continuous stirring, which was maintained for 30 min at 600 rpm. The CS/GlcNAc solution was transferred to a syringe (20 mL capacity and 1 mm diameter outlet tip) and then pumped into the coagulation bath (300 mL—70% aqueous solution of 1M NaOH and 30% methanol; pH 13) [44] at a fixed flow rate (45 mL/h) [45] using a syringe pump (Pump 11 Pico Plus Elite, Harvard Apparatus, Holliston, MA, USA). The filaments formed immediately and entangled randomLy. The distance from the spinneret to the bottom of the coagulation bath container was fixed at 8.6 cm, and all of the experiments were performed at room temperature. Finally, the filaments were taken out, washed with distilled water until the wash water reach a pH close to 7, and subsequently were dried in oven at 65 °C for 1 h. The blank filaments were fabricated by the same method without GlcNAc incorporation. The schematic representation of the fabrication of the CS and GlcNAc-loaded CS filaments is shown in Figure 1. 

### 2.3. Characterization of the Filaments

#### 2.3.1. Scanning Electron Microscopy (SEM)

The surface and cross-section morphology of the CS and CS/GlcNAc wet-spun filaments were observed using a scanning electron microscope Phenom World, Pro-X800-07334 (Eindhoven, The Netherlands) Filament samples were placed on double-sided carbon tape and were coated with a thin layer of gold. The cross-section of the filaments was obtained by fracturing the filament perpendicular to the filament axis under liquid nitrogen. The images were taken by applying an electron beam accelerating voltage of 15 kV, with a depth of focus of 1 mm and a resolution of 30 nm. The average filament diameter (n = 10) and the average pore size were measured using Image J software (Java 1.8.0.112 Version, National Institutes of Health and the Laboratory for Optical and Computational Instrumentation, Wisconsin, WI, USA)). 

#### 2.3.2. XRD Analysis

X-ray diffraction (XRD) profiles were recorded on a Shimadzu model XRD-7000 (Shimadzu, Tokyo/Kyoto, Japan) diffractometer with a Ni-filtered Cu-Kα radiation. The XRD profiles were collected in the scattering range of 2θ = 10–40°, with a resolution of 0.02°, at a scanning speed of 1°/min. The analyses were performed by applying 40 kV of voltage and 30 mA of current. The samples were drawn and arranged in parallel, then submitted to the assay. To evaluate the change of crystallinity in the filaments of CS and CS/GlcNAc, the crystallinity index (CI) was calculated. We performed the deconvolution of the X-ray diffraction patterns and the data and peak fitting. The peaks were fitted with Gaussian functions using nonlinear least squares fitting, with Origin Pro 9.0 software (OriginLab Corporation, Northampton, Massachusetts, MI, USA). The crystallinity index of the samples was calculated by the following equation:

(1) $$\mathrm{CI}(\%)=\frac{A_{\mathrm{cr}}}{A_{\mathrm{sample}}}\times 100$$

where A_cr_ is the area of the crystalline peaks and A_sample_ is the area under the sample intensity curve. 

#### 2.3.3. Mechanical Properties

**Figure 2:**
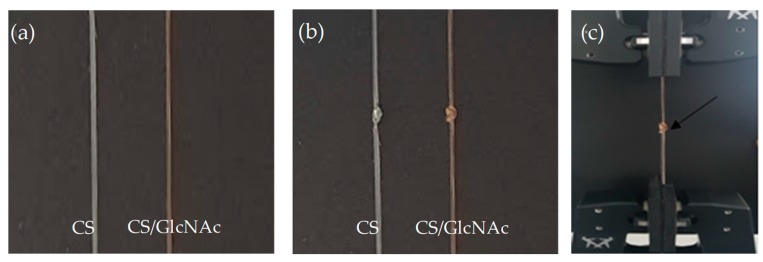
Filaments of CS and CS/GlcNAc: non-knotted (**a**); knotted; (**b**) and placed in the Instron machine with the knot approximately midway between the clamps (**c**).

Uniaxial tensile testing was used to characterize the mechanical properties of the CS and CS/GlcNAc filaments. Experiments were conducted on a mechanical properties testing apparatus (Instron Model 6633) (Norwood, MA, USA), equipped with a load cell of 500 kN, under ambient conditions, where the temperature was 24 ± 2 °C with a relative humidity of 60% ± 2%. The filaments, non-knotted (Figure 2a) and knotted (Figure 2b), were tested in a dry condition (dry), and under incubation conditions (wet), with the filament soaked for 5 min in PBS at 37 °C, so as to mimic the in vivo conditions, and the tests carried out 2 min after removing the filaments from the fluid, according to the United States Pharmacopeia absorbable suture testing standards. Each filament was subjected to tensile forces at a separation rate of 2 mm/min until failure occurred, according to ASTM D2256 standard. Failure was defined as the breakage of the filament, and the tensile strength or tension at failure was defined as the tensile force (N) measured at failure. The resulting load–displacement data collected by the digital acquisition system were converted to stress–strain data in order to calculate the tensile force (N), percentage strain to failure, and elastic modulus. All of the knots were tied wearing surgical gloves, and they were tied by a single investigator over multiple sessions so as to avoid any variation in the knot strength because of surgeon fatigue. The filaments were placed in the Instron machine with the knot approximately midway between the clamps (Figure 2c). The data are expressed as mean ± standard deviation for ten determinations. The data were evaluated using analysis of variance (ANOVA), and the significance of the model was verified with the t-test, with a significance level of 95% (*p* < 0.05) using Action Stat Quality^®^ software (3.5.152.34 Version, EstatCamp Company, São Carlos, Brazil). 

#### 2.3.4. In Vitro Biodegradation

The CS and CS/GlcNAc filaments degradation was investigated by monitoring the mass loss and morphology changes of the filaments by SEM, cut into equal sizes (5.0 cm), as a function of exposure time to a phosphate buffered saline (PBS) or PBS–lysozyme solution. The mass loss measurement was carried out according to ASTM F1635-04. Therefore, the CS and CS/GLcNAc filaments were accurately weighed (M_i_), placed in a solution of PBS or PBS–lysozyme (1.5 μg/mL) at pH 7.4, and incubated at 37 °C. At regular intervals (28, 35, 42, and 49 days), the filaments were taken out from the PBS or PBS/lysozyme solution, rinsed with distilled water, quickly placed on absorbent paper to remove surface water, and weighted (M_t_). The percent of degradation of the filaments was determined by the following formula:

(2) $$\mathrm{Mass}\;\mathrm{loss}=\frac{M_{i}-M_{t}}{M_{i}}\times 100\%$$

where M_i_ is the initial weight and M_t_ is the weight after time t. Each biodegradation experiment was repeated three times, and the percent of biodegradation was expressed as means ± standard deviations. 

#### 2.3.5. In Vitro Drug Release 

The amount of N-acetyl-D-glucosamine (GlcNAc) released from the prepared CS filaments was measured using an ultraviolet-visible (UV-VIS) spectrophotometer (Shimadzu, Model 1800, Kyoto, Japan), and the DDSolver [46], a complementary program for modeling and comparing drug dissolution profiles, was used to evaluate the model of the release system. Also, the λmax values for the absorbance of GlcNAc in PBS (pH = 7.4) were determined by using a UV-VIS spectrophotometer. Briefly, a filament sample (36 cm) was incubated in a 50 mL PBS solution (pH = 7.4) and maintained at 37 ± 0.5 °C. An aliquot of the release medium (3 mL) was withdrawn through a sampling syringe attached with 0.45 μm membrane filter (Millipore, Bedford, MA, USA) at predetermined time intervals (1, 24, 192, 336, 504, 672, 840, and 1176 h), and an equivalent amount of fresh dissolution medium, which was pre-warmed at 37 °C, was replaced. The collected samples were then analysed for GlcNAc content by measuring the absorbance at 192 nm (λmax of GlcNAc in phosphate buffer solution pH 7.4) using a UV spectrophotometer (Shimadzu 1800, Kyoto, Japan). The experiments were performed in triplicate for each filament formulation in an identical manner, in order to minimize the error variation. The average values were used for further data treatment and plotting. The drug concentration was calculated according to a standard curve, and the cumulative release was obtained by the following equation:

(3) $$\mathrm{Cummulative}\;\mathrm{release}\;(\%)=\frac{\sum_{i=0}^{n}C_{i}V_{0}\times 100}{m}$$

where V_0_ is the sampling volume (3.0 mL), C_i_ is the concentration (mg/L) of the release drug collected at time t, and *m* is the mass of the drug incorporated in the polymer (0.8 mg). 

#### 2.3.6. In Vitro Cytotoxicity Studies

An agar diffusion assay was used to evaluate the cytotoxicity of the CS and CS/GlcNAC filaments, according ISO 10993-5 [31]. Extracts were prepared from the filaments of CS and CS/GlcNAc in the proportion of 0.2 g/mL extractive solvent (ultrapure water), and the solvent extractors with the respective samples were autoclaved at 121 °C ± 2 °C for 1 h. The filter papers with an area of 100 mm^2^ (10 mm × 10 mm) were soaked with the extracts. For the cytotoxicity experiments, L929 cells, obtained from the Rio de Janeiro Cell Bank and preserved at the Northeastern Biomaterials Evaluation and Development Laboratory (CERTBIO; Campina Grande-PB, Brazil), were cultured in a six-well plate with a 35 mm diameter, in an RPMI 1640 medium containing 10% fetal bovine serum (FBS), in humidified ovens at a constant temperature of 37 °C ± 1 °C in a 5% ± 1% CO_2_ atmosphere. Cell suspensions with concentrations in the 1.1 to 1.3 × 105 cells/mL, in a 5 mL volume, were seeded for 24 h. After this period, the cultures that presented a uniform cell monolayer and confluency greater than 80% were used for the assay. Then, the medium was replaced with 1 mL of a prepared agar medium containing 1.8% of agar and 0.01% of neutral red solution e MEM (Minimum Essential Medium) 2× concentrated. After the solidification of the agar (10 min), filter papers soaked with the extracts of the filaments (CS and CS/GlcNAc) were placed on the agar surface, as well as the positive (latex) and negative controls (filter paper Whatman n1), which were placed in the center of each plate, and the samples were made in duplicate. After 24 h of incubation, the decolorization index (halo formation) and lysis index were assessed using an optical microscope. The cell lysis was defined as a loss of cell membrane integrity, visible under a light inverted microscope (NIKON TS100 digital, (Tóquio, Japão) with the image magnification resource of NISElements software (3.2 Version, Melville, NY, USA). The cell lysis is scored as Table 1. 

### 2.4. Statistical Analysis

Data were expressed as mean ± standard deviation. The difference in mean values for the mechanical properties and in vitro biodegration were compared using one-way variance analysis (ANOVA), followed by t-test analysis. The level of significance was considered when *p* < 0.05. 

## 3. Results and Discussion

### 3.1. Filament Morphology

**Figure 3:**
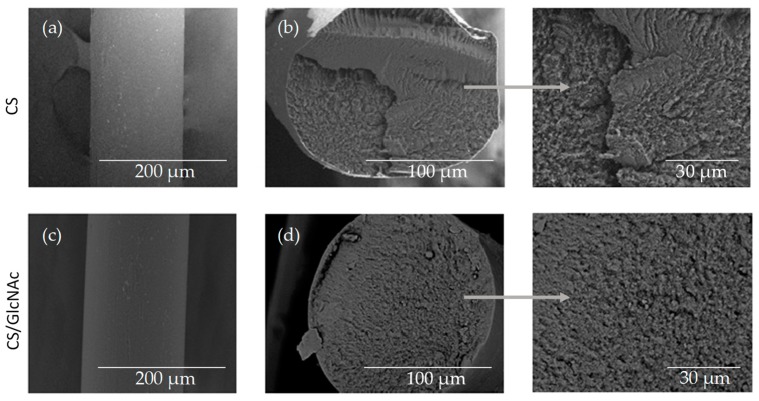
SEM micrographs of the surface (**a**,**c**) and the cross-section (**b**,**d**) morphology of the CS and CS/GlcNAc wet-spun filaments.

**Figure 4:**
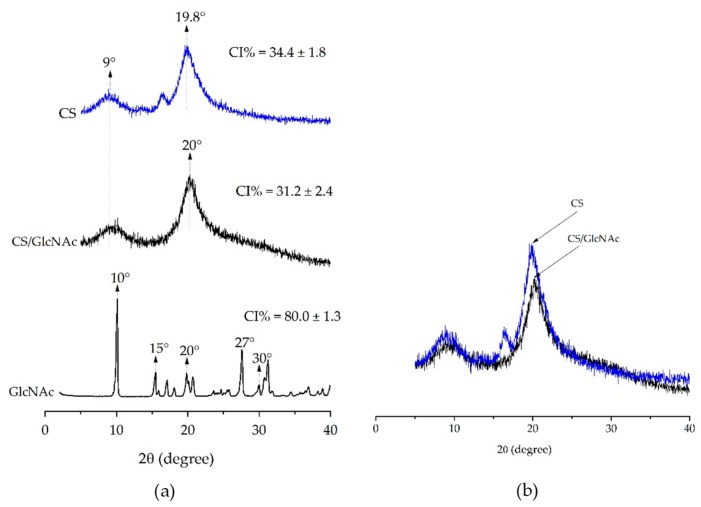
XRD patterns of (**a**) the GlcNAc, CS, and CS/GlcNAc wet-spun filaments, and a (**b**) comparison of the intensity values.

Scanning electron microscopy (SEM) was used to study the surface and cross-sectional morphology of the blank filament (CS) and drug-loaded filament (CS/GlcNAc). Figure 3 shows that both the CS and CS/GlcNAc filaments displayed a smooth, compact, and homogeneous surface topography (Figure 3a,c) and skin–core structure (Figure 3b,d). The skin is a dense layer formed by rapid filament surface coagulation, and the core is a porous structure that is formed once the solvent and nonsolvent are evaporated after filament solidification [48]. In this case, there was a slow coagulation speed inside the filament. This kind of structure is common in filaments fabricated by wet-spun [49]. Indeed, the incorporation of the GlcNAc drug had no significant effect on the shape and cross-sectional morphology. The diameters and average pore size had a slight change after drug incorporation. The average filament diameters of the CS and CS/GlcNAc samples were 145 μm and 148 μm, and the average pore sizes were 5.5 ± 0.2 μm and 4.9 ± 0.2 μm, respectively. The surface and cross-section morphology of the CS and CS/GlcNAc filaments under incubation conditions (in PBS at 37 °C for 5 min) were also investigated. According to the micrographs (not shown here), these filaments likewise displayed a smooth, compact, and homogeneous surface topography and skin–core structure, and the diameters did not changed more than 6.0 µm after incubation in PBS. The average filament diameters of the CS and CS/GlcNAc under incubation conditions were 151 μm and 152 μm, respectively. The results of this characterization also show that the GlcNAc drug was embedded in the filaments, as it was not possible to observe the presence of non-solubilized GlcNAc crystals on the filament samples (Figure 3d). This is believed to be because within the chitosan, GLcNAc is immobilized by combinations of covalent, ionic, and hydrogen bonding interactions [50,51,52], which is corroborated by the XRD results (Figure 4). 

### 3.2. XRD Analysis

Figure 4 shows the XRD patterns of the GlcNAc drug, CS filament (CS), and GlcNAc-loaded CS filament (CS/GlcNAc). In the XRD of the GlcNAc powder, sharp peaks at a diffraction angle of 2θ = 10°, 15°, 15°, 20°, 27°, and 30° were present, which suggest that the drug is present as a crystalline material (Herdyastuti and Cahyaningrum (2017)). However, the absence of GlcNAc specific diffraction in the X-ray diffractogram of drug-loaded CS filaments (CS/GlcNAc) indicates that GlcNAc is present as either an amorphous drug or a solid solution within the amorphous regions of the polymer (molecular dispersion). Lavin et al. found similar results with the anti-inflammatory drug’s, dexamethasone, incorporation into poly(L-lactic acid) microfibers prepared by the wet-spun method [53]. It was also observed that the chitosan loaded with the drug (CS/GLcNAc) had a less ordered state than the chitosan without the drug (CS), and showed peaks with less intensity at a 2θ = 10° and ≈ 20° (Figure 4b), typical fingerprints of chitosan related to hydrated and anhydrous crystals, respectively [54]. The crystallinity index (CI) of the former was 31.2 ± 2.4 and the latter was 34.4 ± 1.8, distinguishing them statistically with a *p*-value < 0.05. 

### 3.3. Mechanical Test

Table 2 presents the mechanical properties data of the blank filaments (CS) and drug-loaded filaments (CS/GlcNAc), non-knotted and knotted, tested under the dry condition (after drying in oven at 65 °C for 1 h; dry) and under incubation conditions (after incubation for 5 min in PBS at 37 °C; wet). This characterization was carried out with the aim to evaluate the potential of these filaments as a suture material. The mechanical properties of the sutures are one of the most important properties, as they determine whether the sutures could keep the wound closed until the tissue heals, without breaking. They must withstand the mechanical strength required during their use and handling. These important characteristics determine the functional performance of this type of material. 

The presence of a knot resulted in a lower breaking strength, Young modulus, and stress at break values for all of the filaments, distinguishing them as statistically with a *p*-value < 0.05, when tested under the same conditions (Table 2). The filaments failure occurred at the knot or at a point near that region, indicating that the knot region was the weakest point of the filament. This can be attributed to a reduction in the diameter of the filament and the high-stress concentration near the knot region [55,56,57]. Lapotinte et al. [9] reported that, regardless of the configuration of the knot, the weakest point of a surgical suture is the knot, and the break in the filament always occurs at a point near that region, which is the most common defect in suture threads. Moreover, the reduction in tensile strength may be 35–95%, depending on the suture material used [7,58]. 

The effect of incubation (wet condition) on the on the mechanical properties of the filaments was also evaluated. According to Table 2, all of the filaments subjected to tensile test after incubation showed a lower breaking strength, Young modulus, and stress at break, and higher elongation values, distinguishing them as statistically with a *p*-value < 0.05, as compared with the filaments tested under the dry condition. Water molecules can easily permeate into the fiber, increasing the filament flexibility as a result of the decrease in hydrogen bonds between the amorphous chains, decreasing the chain packing, which in turn leads to a loss of rigidity, increasing the filament’s maximum elongation values [59,60]. Similar results were reported by Notin et al. [61], who observed that the resistance of the chitosan filaments was sensitive to the presence of moisture, that is, when the hydration of the system increased, the elongation increased and the Young modulus and the stress at break decreased. Moreover, as mentioned previously, the average filament’s diameter under incubation conditions (wet condition) was higher than under the dry condition (see values in Section 3.1), so the cross-sectional area for the former is higher and this increase in the cross-sectional area decreases the stress. 

Besides good handling characteristics and knot security, elongation rate is another important suture property. It is determined by both the elasticity and structural properties of the suture material. The elasticity in the threads allows for the suture to be able to stretch as the wounds swell, and then regain their original shape and length as the swelling decreases. This maintains the position of the tissue during healing, and the wound edges close together. A good suture should still be pliable so as to facilitate handling [50,62,63]. 

Table 2 also shows that the immobilization GlcNAc on chitosan filaments reduced their mechanical properties (particularly for the samples tested under the dry condition). The reason for this may be due to a reduction in the chitosan crystallinity when the GlcNAc was incorporated (Figure 4), caused by the elimination of some inter-chain hydrogen bonds as a result of the interactions between the chitosan and GlcNAc groups. Albanna et al. (2013) [64] showed that the covalent immobilization of heparin onto chitosan fibers caused fiber swelling and a reduction in the fiber mechanical properties, a finding that is consistent with our results for immobilized GlcNAc on chitosan. 

Although the mechanical resistance of the chitosan filaments has decreased with the incorporation of the GlcNAc drug, this property was greater than the mean values indicated in the U.S. Pharmacopeia (1.7 N) for suture number 6-0 (filament diameter of 100–149 μm). Thus, with regard to the mechanical performance, these results indicate that the GlcNAc-loaded CS filaments could be adequate as a material for surgical sutures, and represent a breakthrough in the sense of combined mechanical properties and therapeutic action. 

### 3.4. In Vitro Biodegration

**Figure 5:**
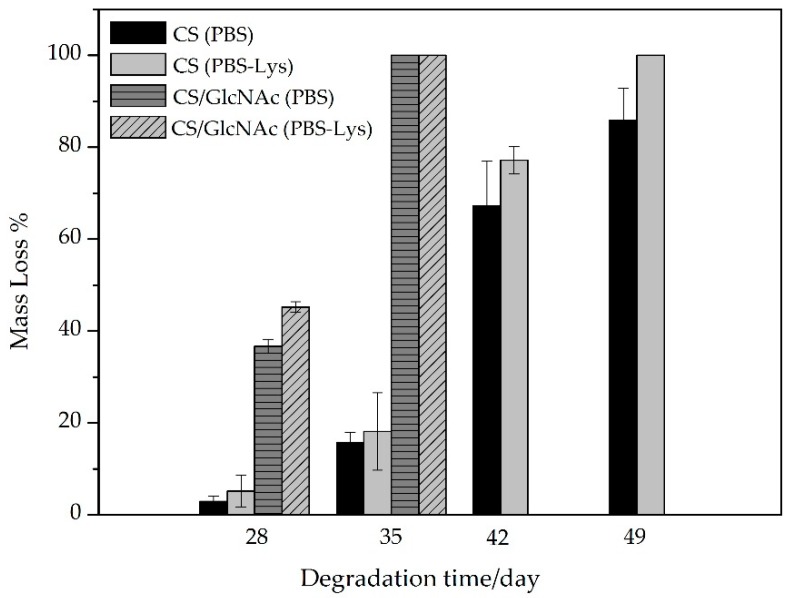
Mass loss of CS and CS/GlcNAc filaments as a function of the degradation time.

For applications of chitosan as a absorbable suture material, the lysozyme degradation rates at the physiological pH-values are of great importance.Thus, the degradation behavior of the CS and CS/GlcNAc filaments was evaluated by measuring the mass loss and morphology changes, as they were degraded in the PBS and PBS–lysozyme (PBS–Lys) solutions separately (pH 7.4) at 37 °C. Based on Figure 5, the filaments in the PBS or PBS–lysozyme solution showed mass loss with increasing degradation time. The mass loss of the filaments in PBS–lysozyme was larger than that of the filaments in PBS solution. These results revealed that the enzyme accelerates the degradation of the filaments [65,66,67,68,69,70]. 

Furthermore, the mass loss of the CS/GlcNAc filaments was larger than that of the CS filaments, distinguishing them statistically with *p*-value < 0.05. After degradation for 28 days, the mass loss of the CS filaments was 2.9 ± 1.1% and 5.2 ± 3.4 in PBS solution and PBS–lysozyme, respectively, whereas the CS/GlcNAc filaments was 36.7 ± 1.4% and 45.3 ± 1.1 in PBS and PBS–lysozyme solutions, respectively, indicating their much faster degradation. After 35 days of degradation either PBS solution or PBS–lysozyme, the CS/GlcNAc filaments degradability was total. On the other hand, only after 49 days, the entire degradation of CS filaments in PBS–lysozyme was noticed. However, in PBS solutions, the mass loss for this filament was 85.8 ± 6.9%. This might be due the fact that GlcNAc caused more amorphous regions in chitosan molecules (Figure 4), which would be more water permeable and in turn more accessible to lysozyme. These results are in accordance with the reported by Sun et al. [71]. 

**Figure 6:**
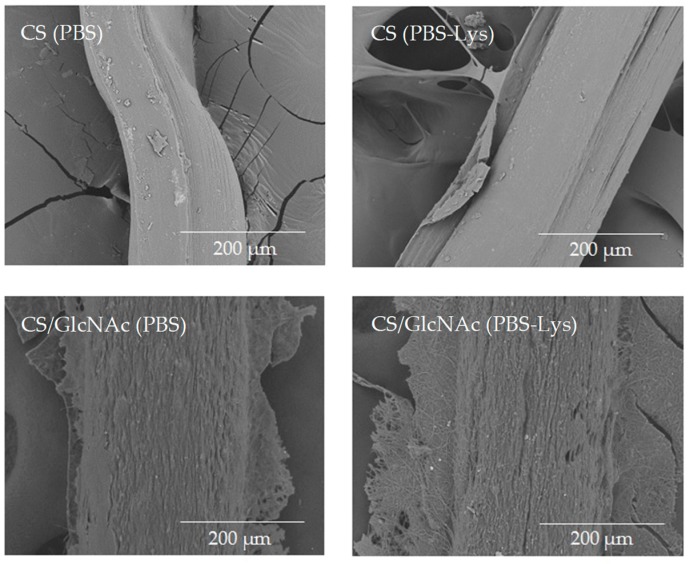
SEM images of the CS and CS/GlcNAc filaments after incubation in a phosphate buffered saline (PBS) solution and PBS–lysozyme (PBS–Lyz) at 37 °C for 49 and 35 days, respectively.

The surface of CS and CS/GlcNAc filaments after 49 and 35 days, respectively, in both degradation media (PBS and PBS–lysozyme solutions) at 37 °C was imaged using SEM to investigate any morphological changes due degradation (Figure 6). After these periods, the filaments had their sizes reduced from 5 cm to values near to 1 mm; this indicated the degradation was finished (i.e., the filaments degradability was complete). The degraded filaments’ SEM micrographs showed that the some lose their shape and displayed a rough surface, especially the CS/GlcNAc filaments in the PBS–lysozyme solutions. These results revealed that the GlcNAc drug accelerates the degradation of the CS filaments. As described previously, this might be due the fact that GlcNAc caused more amorphous regions in the chitosan molecules (Figure 4), which would be more water permeable, and in turn, more accessible to lysozyme. 

Chitosan degradation occurs in the human body, mainly by enzymatic depolymerization through the hydrolysis promoted by lysozyme (which is an enzyme present in the body) of the glyosidic bonds present in the chemical structure of chitosan [67,72]. The enzymatic degradation of chitosan generally leads to a release of monosaccharides, which can be incorporated into the metabolic pathways of glycosaminoglycan and glycoprotein [73]. Chitosan depolymerization compounds are biocompatible, and do not cause either significant inflammation or tissue damage [74,75,76,77]. These results indicated that GlcNAc-loaded CS filaments might be used as a absorbable surgical suture materials. 

### 3.5. In Vitro Drug Release

**Figure 7:**
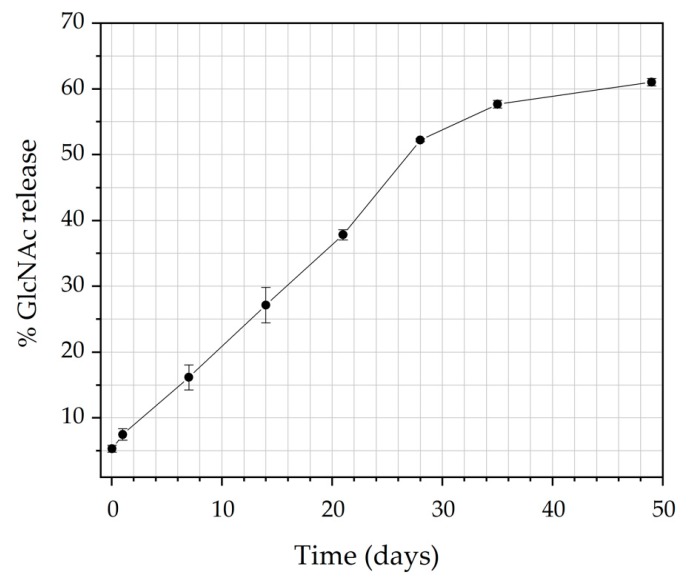
The cumulative release curve of GlcNAc from CS filaments in a pH 7.4 buffer at 37 °C.

The in vitro release behavior of N-acetyl-D-glucosamine (GlcNAc) from CS filaments was assessed at 37 °C in phosphate buffered saline (PBS) as a simulation of the body fluid at a pH of 7.4. Figure 7 shows the percent release of GlcNAc from CS filaments against incubation time. As can be noted from this figure, 7.4% of the GlcNAc was released within one day, and 16.1% within seven days. The relatively low “burst effect” of these filaments is presumably due to the preferential location of the GlcNAc molecules within the deep sections of the CS filaments. As showen in the SEM images presented previously (Figure 3), the GlcNAc crystals could not be observed on the CS filament surface and cross-sectional. Moreover, the strong interaction between CS-GlcNAc, as suggested by the XDR data (Figure 4), may have resulted in a slow desorption of the drug from the filament, and this can reduce the initial burst release effect of the drug. The advantage of this liberation system is avoiding negative consequences, such as the local toxicity from high drug concentrations [78]. Notice that a more pronounced release takes place after 21 days. Indeed, until 21 days, only the swelling of the CS filaments occurs (at 7, 14, and 21 days, the mass gain was 128.4%, 116.5%, and 112.8%, respectively, for the CS filaments in PBS, and was 113.0%, 135.6%, and 101.5% for the CS/GlcNAc filaments), allowing for the aqueous solution to penetrate through the chitosan, resulting in the diffusion of GlcNAc to the medium. After 21 days, as a result of the extended contact of the polymeric matrix with the surrounding medium, the polymer degradation starts to take place (Figure 5), allowing for the remaining drug to be released. About 61.0% of the loaded drug from the CS filaments was released in 49 days. 

Qian et al. [79] described an effective approach for improving glucosamine intestinal absorption, and hence its oral bioavailability, using chitosan. The authors demonstrated, using in vivo studies in rats and beagle dogs, that the presence of chitosan could increase the plasma concentration and bioavailability of glucosamine, without altering its elimination. Thus, GlcNAc-loaded CS filaments can be proposed as promising absorbable surgical suture materials, as it could potentially provide an improved therapeutic effect in comparison with the conventional dose, in view of getting the delivery of the drug in a controlled and prolonged manner. 

### 3.6. Kinetics of Release

The kinetic of the GlcNAc release from the CS filaments was evaluated according to zero-order, Higuchi, Peppas–Sahlin, and Hopfenberg models [80,81,82], using the DDSolver [46], an add-in program for modeling and comparison of drugs [50]. The criteria for selecting the most appropriate model were based on the best goodness of fit of the experimental results, that is, based on the statistically higher values of the adjusted coefficient of determination (r^2^), the lower value of Akaike Information Criterion (AIC), and the largest value of model selection criterion (MSC), the most popular criteria in the field of dissolution model identification [46]. These values are calculated and compared in Table 3. Notice that the GlcNAc release from the CS filaments can be best described using the zero-order and Hopfenberg models, where higher values of r^2^, lower values of AIC, and higher values of MSC were recorded (Table 3). A linear relationship was obtained, suggesting that the GlcNAc release is concentration independent, a situation desirable in the sustained release formulation, because it minimizes the oscillations of drug concentration in the blood [83]. 

It is possible that the release of GlcNAc from the chitosan filaments presents a sustained release dosage, allowing for the drug to remain within the therapeutic range for an extended period of time. This result corroborates with the biodegradation test (Figure 5), where the first stage of the degradation process was found to be within 21 days, which involves swelling without a loss of weight. During this time, the release is probably controlled by a diffusion process, which results in a slower release, because the fluid that occupies the polymer matrix fills the pores, which may eventually facilitate the release of the drug over time. After 28 days, a higher percentage of GlcNAc release occurs, supposedly as a result of the erosion process, a characteristic of the Hopfenberg model, corroborating with the biodegradation test, where the mass loss starts, during this period. Hence, when the system can no longer maintain the integrity of the polymer network, the drug is released more effectively [84]. According to Siegel and Rathbone [85], one characteristic of erosion is that the size of the system decreases with time, and the drug release correlates with erosion (as seen in the SEM images in Figure 6). 

### 3.7. Cytotoxicity Assay

**Figure 8:**
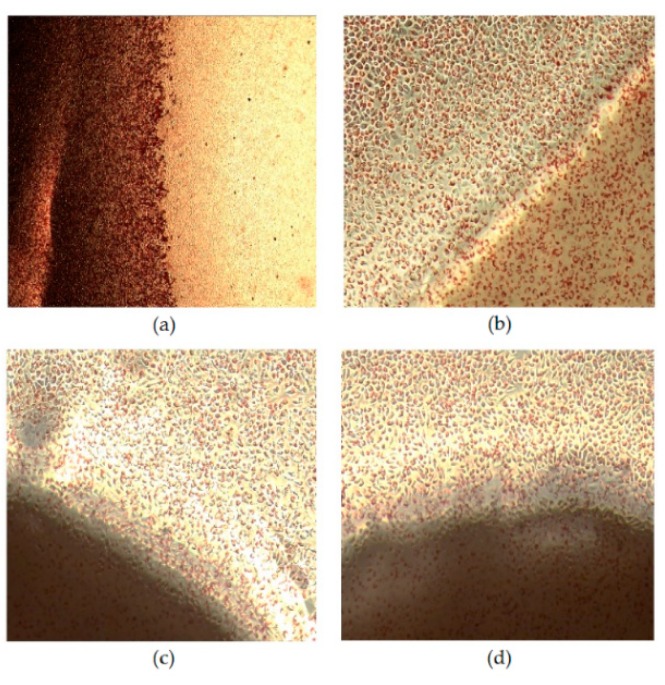
Phase contrast microscopy images of L929 cells after incubation for 24 h: (**a**) positive control, (**b**) negative control, (**c**) CS filament, and (**d**) CS/GlcNAc filament. All of the figures are of the same magnification.

The in vitro cytotoxicity tests of the CS and CS/GlcNAc filaments toward L929 cells were evaluated using the agar diffusion method. The cytotoxicity was assessed by observing the size of the halo around the tested material after 24 h of incubation, using the scoring criteria presented in Table 1. From the evaluation of the halos, the qualitative results of the positive control, negative control, and the CS and CS/GlcNAc filaments were obtained, and the mean values found for the samples are shown in Table 4. As shown in Figure 8a, there was the formation of a halo around the positive control, revealing cell death (lysis) [86] (i.e., their toxicity). As established by ISO 10993-5 (Table 1), for the size of the halo between 0.45 and 1.0 cm, the cytotoxicity is moderate (grade 3). Thus, once the size of the bleaching zone of the positive control was 0.92 cm (Table 4), their cytotoxicity was moderate. The results of the cytotoxicity tests with the CS and CS/GlcNAc filaments showed that the L929 cell line did not present a halo formation around the filaments, and changes in the cell morphology were not observed (Figure 8c,d), similarly to the negative controls (Figure 8b). The obtained results clearly suggest that the CS and CS/GlcNAc filaments are nontoxic to L929 cells, and enables their potential use as an absorbable surgical suture material. These findings are in agreement with the previously reported data attesting to the non-cytotoxicity of chitosan-based materials [87,88,89,90,91,92,93]. 

## 4. Conclusions

Chitosan and of N-acetyl-D-glucosamine-loaded chitosan filaments were fabricated by the wet-spinning method, with a smooth, compact, and homogeneous surface topography and skin–core structure, with average filament diameters of 145 µm and 148 µm, respectively. The immobilization of N-acetyl-D-glucosamine (GlcNAc) on the chitosan (CS) filaments reduced their mechanical properties. However, it was greater than the mean values indicated in the U.S. Pharmacopeia for surgical sutures. The biodegradation of the CS filaments was accelerated by the addition of GlcNAc. The in vitro kinetic of the release process was of the apparent zero-order and Hopfenberg models type, controlled by both the diffusion and erosion process. Thus, the GlcNAc release from the CS filaments was concentration independent, a situation desirable in the sustained release formulation. The in vitro cytotoxicity data of the CS and CS/GlcNAc filaments toward the L929 cells showed that these filaments are nontoxic to these cells. Thus, the mechanical properties of the GlcNAc-loaded CS filaments, along with the other characteristics, such as the biodegradation, biocompatibility, and prolonged release of the drug, made it a desirable candidate for absorbable surgical suture material applications. Moreover, this medical device may be able to enhance healing processes, relieve pain, and minimize infection at the surgery site as a result of the prolonged release of GlcNAc. Studies on the GlcNAc release in vivo and its healing response are going on and will be reported on further. 

## Figures and Tables

**Table 1:** International Organization of Standardization (ISO) 10993-5:2009 criteria for reactivity scoring of the agar diffusion assay [47].

Grade	Reactivity	Reactivity Zone Description
0	None	No detectable zone around or under the specimen
1	Slight	Some malformed or degenerated cells under the specimen
2	Mild	Zone limited to the area under the specimen
3	Moderate	Zone extending the specimen size up to 1.0 cm
4	Severe	Zone extending farther than 1.0 cm beyond the specimen

**Table 2:** Mechanical properties of the chitosan (CS) and N-acetyl-D-Glucosamine (GlcNAc)-loaded CS filaments—non-knotted and knotted—tested in a dry condition (dry) and under incubation conditions (wet).

Filament Type	Sample	Breaking Strength (N)	Elongation at Break (%)	Young Modulus (MPa)	Stress at Break (MPa)
Non-knotted	CS Dry	4.3 ± 0.5	8.9 ± 1.1	25.2 ± 0.1	261.0 ± 25.1
	CS Wet	2.9 ± 0.5	15.9 ± 5.3	6.2 ± 0.3	170.9 ± 29.8
	CS/GlcNAc Dry	3.4 ± 0.7	8.6 ± 1.0	21.7 ± 2.0	181.9 ± 71.5
	CS/GlcNAc Wet	2.8 ± 0.8	17.2 ± 3.9	7.2 ± 0.2	159.4 ± 39.0
Knotted	CS Dry	2.4 ± 0.5	10.5 ± 4.3	17.8 ± 0.2	127.1 ± 32.8
	CS Wet	2.7 ± 0.7	11.4 ± 2.8	1.2 ± 0.3	104.4 ± 22.3
	CS/GlcNAc Dry	1.9 ± 0.2	9.2 ± 2.6	9.1 ± 0.2	95.4 ± 26.1
	CS/GlcNAc Wet	1.8 ± 0.7	16.6 ± 5.4	4.8 ± 1.4	88.3 ± 51.1

**Table 3:** Fitting results of the experimental GlcNAc release data to different kinetic models.

Models	r^2^	AIC	MSC
Zero-order	0.994	30.52	4.08
Higuchi	0.908	46.08	2.14
Peppas–Sahlin	0.987	35.72	3.43
Hopfenberg	0.981	33.85	3.66

**Table 4:** Cytotoxicity assessment using the agar zone test.

	Reading the Plates Average (cm)	Cytotoxicity Degree
Negative Control	0.00	0
Positive Control	0.92	3
Chitosan	0.00	0
Chitosan/GlcNAc	0.00	0

## References

[1] Chu C.C. (2013). 10—Types and Properties of Surgical Sutures.

[2] Kladakis S. (2015). Choosing Sutures in Small Animal Surgery. J. Dairy Vet. Anim. Res..

[3] Kim J.C., Lee Y.K., Lim B.S., Rhee S.H., Yang H.C. (2007). Comparison of tensile and knot security properties of surgical sutures. J. Mater. Sci. Mater. Med..

[4] Pillai C.K.S., Sharma C.P. (2010). Review Paper: Absorbable Polymeric Surgical Sutures: Chemistry, Production, Properties, Biodegradability, and Performance. J. Biomater. Appl..

[5] Viju S., Thilagavathi G. (2012). Fabrication and characterization of silk braided sutures. Fibers Polym..

[6] Gogoi D., Choudhury A.J., Chutia J., Pal A.R., Khan M., Choudhury M., Pathak P., Das G., Patil D.S. (2014). Development of advanced antimicrobial and sterilized plasma polypropylene grafted MUGA (antheraea assama) silk as suture biomaterial. Biopolymers.

[7] Greenberg J.A., Clark R.M. (2009). Advances in suture material for obstetric and gynecologic surgery. Rev. Obstet. Gynecol..

[8] Debus E.S., Geiger D., Sailer M., Ederer J., Thiede A. (1997). Physical, Biological and Handling Characteristics of Surgical Suture Material: A Comparison of Four Different Multifilament Absorbable Sutures. Eur. Surg. Res..

[9] Lapointe S., Zhim F., Sidéris L., Drolet P., Célestin-Noël S., Dubé P. (2016). Effect of chemotherapy and heat on biomechanical properties of absorbable sutures. J. Surg. Res..

[10] Carter A., Skilbeck C.J. (2014). Sutures, ligatures and knots. Surgery.

[11] Rethinam S., Thotapalli Parvathaleswara S., Nandhagobal G., Alagumuthu T., Robert B. (2018). Preparation of absorbable surgical suture: Novel approach in biomedical application. J. Drug Deliv. Sci. Technol..

[12] Nakajim M., Atsum K., Kifune K., Miura K., Kanamaru H. (1986). Chitin is an Effective Material for Sutures. Jpn. J. Surg..

[13] Montenegro R., Gordeiro J.R.G. (2012). Chitosan Based Suture Focusing on the Real Advantages of an Outstanding. Adv. Chitin Sci..

[14] Cheung R.C.F., Ng T.B., Wong J.H., Chan W.Y. (2015). Chitosan: An update on potential biomedical and pharmaceutical applications. Mar. Drugs.

[15] Prudden J.F., Balassa L., Friedrich L., Hanson P., Migel P. (1970). The discovery of a potent pure chemical wound-healing accelerator. Am. J. Surg..

[16] Vert M., Hellwich K.H., Hess M. (2012). Terminology for biorelated polymers and applications. Pure Appl. Chem.

[17] Lee J.E., Park S., Park M., Kima M.H., Park C.G., Lee S.H., Choi S.Y., Kim B.H., Park H.J., Park J.-H. (2013). Surgical suture assembled with polymeric drug-delivery sheet for sustained, local pain relief. Acta Biomater..

[18] Shen Z., Kamdem D.P. (2015). Development and characterization of biodegradable chitosan films containing two essential oils. Int. J. Biol. Macromol..

[19] Wang J., Wang L., Yu H., Zain-ul-Abdin, Chen Y., Chen Q., Zhou W., Zhang H., Chen X. (2016). Recent progress on synthesis, property and application of modified chitosan: An overview. Int. J. Biol. Macromol..

[20] Gu B.K., Park S.J., Kim M.S., Lee Y.J., Kim J.-I., Kim C.-H. (2016). Gelatin blending and sonication of chitosan nanofiber mats produce synergistic effects on hemostatic functions. Int. J. Biol. Macromol..

[21] Mohammadi A., Hashemi M., Masoud Hosseini S. (2016). Effect of chitosan molecular weight as micro and nanoparticles on antibacterial activity against some soft rot pathogenic bacteria. LWT Food Sci. Technol..

[22] Szymańska E., Winnicka K. (2015). Stability of chitosan—A challenge for pharmaceutical and biomedical applications. Mar. Drugs.

[23] Liu Z., Jiao Y., Wang Y., Zhou C., Zhang Z. (2008). Polysaccharides-based nanoparticles as drug delivery systems. Adv. Drug Deliv. Rev..

[24] Dash M., Chiellini F., Ottenbrite R.M., Chiellini E. (2011). Chitosan—A versatile semi-synthetic polymer in biomedical applications. Prog. Polym. Sci..

[25] De la Fuente M., Raviña M., Paolicelli P., Sanchez A., Seijo B., Alonso M.J. (2010). Chitosan-based nanostructures: A delivery platform for ocular therapeutics. Adv. Drug Deliv. Rev..

[26] Saber A., Strand S.P., Ulfendahl M. (2010). Use of the biodegradable polymer chitosan as a vehicle for applying drugs to the inner ear. Eur. J. Pharm. Sci..

[27] Park J.H., Saravanakumar G., Kim K., Kwon I.C. (2010). Targeted delivery of low molecular drugs using chitosan and its derivatives. Adv. Drug Deliv. Rev..

[28] Pellá M.C.G., Lima-Tenório M.K., Tenório-Neto E.T., Guilherme M.R., Muniz E.C., Rubira A.F. (2018). Chitosan-based hydrogels: From preparation to biomedical applications. Carbohydr. Polym..

[29] Li J., Liu D., Hu C., Sun F., Gustave W., Tian H., Yang S. (2016). Flexible fibers wet-spun from formic acid modified chitosan. Carbohydr. Polym..

[30] Gokarneshan N., Dhatchayani U. (2018). Performance Evaluation of Newer Types of Silk Surgical Sutures. J. Gerontol. Geriatr. Med..

[31] Casalini T., Masi M., Perale G. (2012). Drug eluting sutures: A model for in vivo estimations. Int. J. Pharm..

[32] Joseph B., George A., Gopi S., Kalarikkal N., Thomas S. (2017). Polymer sutures for simultaneous wound healing and drug delivery—A review. Int. J. Pharm..

[33] Padmakumar S., Joseph J., Neppalli M.H., Mathew S.E., Nair S.V., Shankarappa S.A., Menon D. (2016). Electrospun Polymeric Core-sheath Yarns as Drug Eluting Surgical Sutures. ACS Appl. Mater. Interfaces.

[34] Weldon C.B., Tsui J.H., Shankarappa S.A., Nguyen V.T., Ma M., Anderson D.G., Kohane D.S. (2012). Electrospun drug-eluting sutures for local anesthesia. J. Control. Release.

[35] Mccarty M.F. (1996). Glucosamine for Wound Healing. Med. Hypotheses.

[36] Muzzarelli R.A.A. (1997). Human enzymatic activities related to the ther- apeutic administration of chitin derivatives. Cell. Mol. Life Sci..

[37] Schoukens G. (2019). Bioactive Dressings to Promote Wound Healing.

[38] Ashkani-Esfahani S. (2012). Glucosamine Enhances Tissue Regeneration In The Process Of Wound Healing In Rats As Animal Model; A Stereological Study. J. Cytol. Histol..

[39] Hyun H., Park M., Jo G., Kim S., Chun H., Yang D. (2019). Photo-Cured Glycol Chitosan Hydrogel for Ovarian Cancer Drug Delivery. Mar. Drugs.

[40] Dalirfardouei R., Karimi G., Jamialahmadi K. (2016). Molecular mechanisms and biomedical applications of glucosamine as a potential multifunctional therapeutic agent. Life Sci..

[41] Brugnerotto J., Lizardi J., Goycoolea F., Argüelles-Monal W., Desbrières J., Rinaudo M. (2001). An infrared investigation in relation with chitin and chitosan characterization. Polymer (Guildf).

[42] Reddy N., Yang Y. (2015). Electrospun Fibers from Polysaccharides. Innov. Biofibers Renew. Resour..

[43] Yudin V.E., Dobrovolskaya I.P., Neelov I.M., Dresvyanina E.N., Popryadukhin P.V., Ivan’Kova E.M., Elokhovskii V.Y., Kasatkin I.A., Okrugin B.M., Morganti P. (2014). Wet spinning of fibers made of chitosan and chitin nanofibrils. Carbohydr. Polym..

[44] Foroughi J., Mirabedini A., Warren H. (2018). Hydrogels Fibers.

[45] Cruz R.d.C.A.L., Diniz L.G.M., Lisboa H.M., Fook M.V.L. (2016). Effect of different carboxylic acids as solvent on chitosan fibers production by wet spinning. Rev. Matér..

[46] Zhang Y., Huo M., Zhou J., Zou A., Li W., Yao C., Xie S. (2010). DDSolver: An Add-In Program for Modeling and Comparison of Drug Dissolution Profiles. AAPS J..

[47] (2009). ISO-10993-5 Biological Evaluation of Medical Devices—Part 5: Tests for in Vitro Cytotoxicity.

[48] Rissanen M., Puolakka A., Nousiainen P., Kellomaki M., Ella V. (2008). Solubility and Phase Separation of Poly(L,D-Lactide) Copolymers. J. Appl. Polym. Sci..

[49] Lavin D.M., Zhang L., Furtado S., Hopkins R.A., Mathiowitz E. (2013). Effects of protein molecular weight on the intrinsic material properties and release kinetics of wet spun polymeric microfiber delivery systems. Acta Biomater..

[50] Dart A.J., Dart C.M. (2017). Suture Material: Conventional and Stimuli Responsive. Compr. Biomater. II.

[51] Madihally S.V., Matthew H.W.T. (1999). Porous chitosan scaffolds for tissue engineering. Biomaterials.

[52] Madihally S.V., Flake A.W., Matthew H.W.T. (1999). Maintenance of CD34 expression during proliferation of CD34+ cord blood cells on glycosaminoglycan surfaces. Stem Cells.

[53] Lavin D.M., Stefani R.M., Zhang L., Furtado S., Hopkins R.A., Mathiowitz E. (2012). Multifunctional polymeric microfibers with prolonged drug delivery and structural support capabilities. Acta Biomater..

[54] Baklagina Y.G., Klechkovskaya V.V., Kononova S.V., Petrova V.A., Poshina D.N., Orekhov A.S., Skorik Y.A. (2018). Polymorphic Modifications of Chitosan. Crystallogr. Rep..

[55] Heward A.G., Laing R.M., Carr D.J., Niven B.E. (2004). Tensile Performance of Nonsterile Suture Monofilaments Affected by Test Conditions. Text. Res. J..

[56] Muffly T.M., Boyce J., Kieweg S.L., Bonham A.J. (2010). Tensile Strength of a Surgeon’s or a Square Knot. J. Surg. Educ..

[57] Greenberg J.A., Goldman R.H. (2013). Barbed suture: a review of the technology and clinical uses in obstetrics and gynecology. Rev. Obstet. Gynecol..

[58] Dart A.J., Dart C.M. (2011). Suture Material: Conventional and Stimuli Responsive. Comprehrnsive Biomater..

[59] Vehoff T., Glišović A., Schollmeyer H., Zippelius A., Salditt T. (2007). Mechanical properties of spider dragline silk: Humidity, hysteresis, and relaxation. Biophys. J..

[60] Judawisastra H., Hadyiswanto I.O.C., Winiati W. (2012). The Effects of Demineralization Process on Diameter, Tensile Properties and Biodegradation of Chitosan Fiber. Procedia Chem..

[61] Notin L., Viton C., David L., Alcouffe P., Rochas C., Domard A. (2006). Morphology and mechanical properties of chitosan fibers obtained by gel-spinning: Influence of the dry-jet-stretching step and ageing. Acta Biomater..

[62] Barros M., Gorgal R., Machado A.P., Correia A., Montenegro N. (2011). Princípios básicos em cirurgia: Fios de sutura. Acta Med. Port..

[63] Alves A.P., Sá M.J.C., Fook M.V.L., Felipe G.C., Henrique F.V., Albuquerque E.E., Medeiros L.K.G., Alexandre P.R.S. (2017). Avaliação biomecânica e dimensional do fio de sutura à base de quitosana. Arq. Bras. Med. Vet. Zootec..

[64] Albanna M.Z., Bou-Akl T.H., Blowytsky O., Walters H.L., Matthew H.W.T. (2013). Chitosan fibers with improved biological and mechanical properties for tissue engineering applications. J. Mech. Behav. Biomed. Mater..

[65] Su B., Sun S., Zhao C., Capri A. (2011). Polyethersulfone Hollow Fiber Membranes for Hemodialysis. Progress in Hemodialysis—From Emergent Biotechnology to Clinical Practice.

[66] AIBA S.-I. (1992). Studies on chitosan: 4. Lysozymic hydrolysis of partially N-acetylated chitosans. Int. J. Biol. Macromol..

[67] Nordtveit R.J., Vhrum K.M., Smidsrod O. (1994). Degradation of fully water-soluble, partially N-acetylated chitosans with lysozyme. Carbohydr. Polym..

[68] Lee K.Y., Ha W.S., Park W.H. (1995). Blood compatibility and biodegradability of partially N-acylated chitosan derivatives. Biomaterials.

[69] Nordtveit R.J., Vårum K.M., Smidsrød O. (1996). Degradation of partially N-acetylated chitosans with hen egg white and human lysozyme. Carbohydr. Polym..

[70] Onishi H., Machida Y. (1999). Biodegradation and distribution of water-soluble chitosan in mice. Biomaterials.

[71] Sun L., Wanasekara N., Chalivendra V., Calvert P. (2015). Nano-Mechanical Studies on Polyglactin Sutures Subjected to In Vitro Hydrolytic and Enzymatic Degradation. J. Nanosci. Nanotechnol..

[72] Laranjeira M.C.M., Fávere V.T. (2009). de Quitosana:Biopolímero Funcional com Potencial Industrial Biomédico. Quim. Nova.

[73] Lizardi-Mendoza J., Argüelles Monal W.M., Goycoolea Valencia F.M. (2016). Chapter 1—Chemical Characteristics and Functional Properties of Chitosan. Chitosan in the Preservation Agricultural Commodities.

[74] Li Q., Dunn E.T., Grandmaison E.W., Goosen M.F.A. (1992). Applications and Properties of Chitosan. J. Bioact. Compat. Polym..

[75] Wang X.H., Cui F.Z., Feng Q.L., Li J.C., Zhang Y.H. (2003). Preparation and Characterization of Collagen/Chitosan Matrices As Potential Biomaterials. J. Bioact. Compat. Polym..

[76] Kean T., Thanou M. (2010). Biodegradation, biodistribution and toxicity of chitosan. Adv. Drug Deliv. Rev..

[77] Kumar  M.N.V.R. (2000). A review of chitin and chitosan applications. React. Funct. Polym..

[78] Huang X., Brazel C.S. (2001). On the importance and mechanisms of burst release in matrix-controlled drug delivery systems. J. Control. Release.

[79] Qian S., Zhang Q., Wang Y., Lee B., Betageri G.V., Chow M.S.S., Huang M., Zuo Z. (2013). Bioavailability enhancement of glucosamine hydrochloride by chitosan. Int. J. Pharm..

[80] Dredán J., Zelkó R., Antal I., Bihari E., Rácz I. (1998). Effect of chemical properties on drug release from hydrophobic matrices. Int. J. Pharm..

[81] Vueba M.L., Batista de Carvalho L.A.E., Veiga F., Sousa J.J., Pina M.E. (2004). Influence of cellulose ether polymers on ketoprofen release from hydrophilic matrix tablets. Eur. J. Pharm. Biopharm..

[82] Sood A., Panchagnula R. (1998). Drug release evaluation of diltiazem CR preparations. Int. J. Pharm..

[83] NAJIB N., SULEIMAN M. (1985). The kinetics of drug release from ethylcellulose solid dispersions. Drug Dev. Ind. Pharm..

[84] Siepmann J., Siepmann F. (2012). Modeling of diffusion controlled drug delivery. J. Control. Release.

[85] Siegel R.A., Rathbone M.J., Siepmann J., Siegel R.A., Rathbone M.J. (2012). Fundamentals and Applications of Controlled Release. Drug Delivery.

[86] Vulcani V.A.S., Bizarria M.T.M., d’Ávila M.A., Mei L.H.I., Bernal C., Perussi J.R. (2012). Cytotoxicity tests for nanostructured chitosan/PEO membranes using the agar diffusion method. Mater. Res..

[87] Rejinold N.S., Muthunarayanan M., Muthuchelian K., Chennazhi K.P., Nair S.V., Jayakumar R. (2011). Saponin-loaded chitosan nanoparticles and their cytotoxicity to cancer cell lines in vitro. Carbohydr. Polym..

[88] Mansouri S., Lavigne P., Corsi K., Benderdour M., Beaumont E., Fernandes J.C. (2004). Chitosan-DNA nanoparticles as non-viral vectors in gene therapy: strategies to improve transfection efficacy. Eur. J. Pharm. Biopharm..

[89] Temtem M., Silva L.M.C., Andrade P.Z., dos Santos F., da Silva C.L., Cabral J.M.S., Abecasis M.M., Aguiar-Ricardo A. (2009). Supercritical CO_2_ generating chitosan devices with controlled morphology. Potential application for drug delivery and mesenchymal stem cell culture. J. Supercrit. Fluids.

[90] Archana D., Dutta J., Dutta P.K. (2013). Evaluation of chitosan nano dressing for wound healing: Characterization, in vitro and in vivo studies. Int. J. Biol. Macromol..

[91] He Q., Ao Q., Wang A., Gong Y., Zhao N., Zhang X. (2007). In Vitro Cytotoxicity and Protein Drug Release Properties of Chitosan/Heparin Microspheres. Tsinghua Sci. Technol..

[92] Xu Y., Han J., Chai Y., Yuan S., Lin H., Zhang X. (2018). Development of porous chitosan/tripolyphosphate scaffolds with tunable uncross-linking primary amine content for bone tissue engineering. Mater. Sci. Eng. C.

[93] Zhao R., Li X., Sun B., Zhang Y., Zhang D., Tang Z., Chen X., Wang C. (2014). Electrospun chitosan/sericin composite nanofibers with antibacterial property as potential wound dressings. Int. J. Biol. Macromol..

